# A Novel Non-Contact Multi-User Online Indoor Positioning Strategy Based on Channel State Information

**DOI:** 10.3390/s24216896

**Published:** 2024-10-27

**Authors:** Yixin Zhuang, Yue Tian, Wenda Li

**Affiliations:** 1 Science and Engineering, Xiamen University of Technology, No. 600, Ligong Road, Jimei District, Xiamen 361024, China; yixinzhuang@outlook.com; 2 School of Engineering and Physical Sciences, Heriot-Watt University, Edinburgh EH14 4AS, UK; wenda.li@hw.ac.uk

**Keywords:** channel state information (CSI), indoor fingerprint positioning, neighborhood component analysis (NCA), K-means, cross-validation (CV), K-nearest neighbor classification (KNN)

## Abstract

The IEEE 802.11bf-based wireless fidelity (WiFi) indoor positioning system has gained significant attention recently. It is important to recognize that multi-user online positioning occurs in real wireless environments. This paper proposes an indoor positioning sensing strategy that includes an optimized preprocessing process and a new machine learning (ML) method called NKCK. The NKCK method can be broken down into three components: neighborhood component analysis (NCA) for dimensionality reduction, K-means clustering, and K-nearest neighbor (KNN) classification with cross-validation (CV). The KNN algorithm is particularly suitable for our dataset since it effectively classifies data based on proximity, relying on the spatial relationships between points. Experimental results indicate that the NKCK method outperforms traditional methods, achieving reductions in error rates of 82.4% compared to naive Bayes (NB), 85.0% compared to random forest (RF), 72.1% compared to support vector machine (SVM), 64.7% compared to multilayer perceptron (MLP), 50.0% compared to density-based spatial clustering of applications with noise (DBSCAN)-based methods, 42.0% compared to linear discriminant analysis (LDA)-based channel state information (CSI) amplitude fingerprinting, and 33.0% compared to principal component analysis (PCA)-based approaches. Due to the sensitivity of CSI, our multi-user online positioning system faces challenges in detecting dynamic human activities, such as human tracking, which requires further investigation in the future.

## 1. Introduction

Indoor positioning systems (IPSs) are utilized to locate users or objects through various network services, including location-based services (LBSs) [1]. These systems leverage different technologies, such as visual [2], ultrasonic [3], geomagnetic [4], and electromagnetic services, which include infrared rays (IR) [5], visible light [6], and radio waves [7,8,9,10,11]. Among these technologies, wireless fidelity (WiFi) [12] has attracted much attention because of the low deployment cost. The WiFi positioning system (WPS) based on fingerprints collects channel state information (CSI) to build the fingerprint database. The need for the easy deployment of signal devices, such as Bluetooth low-energy (BLE) beacons [13], reconfigurable intelligent surface (RIS)-can [14] systems, and smartphones [15], creates logistical challenges in various indoor environments. These challenges can hinder the effectiveness of multi-user positioning solutions.

Related work [16,17,18] has described systems for indoor positioning; however, these methods are unable to recognize the online positioning of multiple users. These solutions primarily focus on single-user positioning, which restricts their applicability in multi-user scenarios. This limitation presents challenges in scalability and efficiency when trying to locate multiple users at the same time. By discussing these limitations in the relevant sections of this paper, we aim to provide a clearer understanding of how they impact the development of multi-user location solutions. We implement a multi-user system using an access point (AP), three collected CSI devices, and three received devices. The collected CSI devices are Raspberry equipped with commodity WiFi NICs. The robustness is validated with extensive experiments in three different environments. The results show that our multi-user system achieves a median localization error of 1.26 m. Additionally, this new machine learning (ML) method is superior to other methods in a multi-user localizing environment. Our contributions can be summarized as follows:

- To identify multi-user online positioning, this paper designs transmission and collection links using multiple Raspberry devices to collect offline fingerprints and realize multi-user online positioning.
- To overcome the disruption of channel fading in collecting WiFi CSI, we propose a data preprocessing method.
- In this paper, a new ML method called NKCK is proposed, which includes neighborhood components analysis (NCA) dimensionality reduction, K-means clustering, and K-nearest neighbor (KNN) classification based on cross-validation (CV).

Section 2 introduces indoor positioning methods and their lack of multi-user online positions. Section 3 gives the positioning system framework including the CSI feature and Fresnel Zone Principle. Section 4 describes the data preprocessing and the new method. Section 5 presents the experimental results. The conclusion of this research is presented in Section 6.

## 2. Related Work

### 2.1. Traditional Indoor Positioning

The CSI indoor positioning system is listed below. Wu et al. [19] utilized principal component analysis (PCA) and support vector machine (SVM) for indoor positioning, focusing on a single transmitter–receiver pair. In contrast, Wang et al. [20] combined PCA with discrete wavelet transform (DWT) in their system, which employed multiple transmitter–receiver pairs. Fang et al. [21] developed a CSI fingerprint model using a multilevel discrete wavelet transform (MDWT), though their approach was limited to a single transmitter–receiver link. Liu et al. [22] proposed a CSI amplitude fingerprinting algorithm based on linear discriminant analysis (LDA) for indoor positioning, but like previous studies, their system also relied on a single transmitter–receiver setup. Song et al. [23] applied multidimensional scaling analysis to calculate the Euclidean distance and time-reversal resonating strength, followed by the KNN algorithm for location estimation. Li et al. [24] optimized CSI data by integrating DWT with PCA and employed SVM to predict CSI features. The NB-IoT system incorporates multiple reference points (RPs) and NB-IoT base stations (NBSTs). However, due to its low power, it struggles to obtain sufficient CSI data, which results in limited positioning accuracy.

### 2.2. CSI Fingerprint Feature Extraction

The CSI fingerprint feature extraction process includes noise elimination and dimensionality reduction. Zhang et al. [17] proposed an innovative long short-term memory (LSTM) network that integrates CSI phase and amplitude data. However, their system utilized only a single AP and receiver, limiting it to single-user positioning without accommodating multi-user scenarios. Zhu et al. [25] used a broad learning system to combat noise interference in the fingerprint database, but their setup also relied on a single transmitter–receiver pair, resulting in single-user positioning. Similarly, Zhou et al. [26] applied density-based spatial clustering of applications with noise (DBSCAN) to minimize noise and PCA to extract key fingerprint features. Their system was also restricted to single-user positioning with a single transmitter–receiver configuration. Zhou et al. [27] combined a tensor decomposition algorithm with the alternating least squares (ALS) iterative algorithm, but their system remained limited to a single link. Liu et al. [28] implemented parallel AdaBoost to develop a set of weak classifiers for indoor positioning, yet their system involved only one transmitter and receiver. Rao et al. [29] introduced the DFPhaseFL system, which incorporated transfer learning and a deep supervised neural network, but it too was confined to a single AP and monitoring device for positioning. Table 1 summarizes the performance of these various methods. Despite achieving good localization performance, these approaches focus primarily on single-user online positioning.

## 3. CSI Feature and Fresnel Zone Principle

### Description and Characteristics of CSI Signal

The significance of CSI lies in its ability to provide detailed insights into the communication channel. This information is crucial for optimizing wireless communication systems in terms of throughput, reliability, and signal strength. In indoor localization systems, CSI is particularly valuable because it captures the unique characteristics of the environment, such as reflections, diffraction, and scattering caused by walls, furniture, and other objects. Channel estimation refers to the process of estimating the CSI within a specific wireless channel model, where CSI describes the properties of the channel in wireless communications.

In this paper, the multi-path propagation model describes wireless characteristic information in a specific space, which reflects the distinction of CSI in different positions. Let us assume that H(f,t),Y(f,t) and X(f,t) are the CSI matrix, input signal, and output signal. They form the Y(f,t)=X(f,t) × H(f,t)+N relationship and *N* is the surrounding noise. This feature can be regarded as the dependency of a certain fingerprint point.

In a linear time-invariant model, the CSI matrix represents the wireless channel impulse response (CIR), which can be described as follows: $\alpha_{p}\left(f_{i},t\right)$, $2\pi f_{i}$, and $\tau_{p}\left(t\right)$ denote the amplitude attenuation, phase offset, and time delay of the i-th path. *N* is the total number of propagation paths and is the Dirac pulse function. The CSI can be described as follows

(1) $$H\left(f_{i},t\right)=e^{-j2\pi \Delta f_{l}}\;\cdot \;\sum_{p=1}^{P}\alpha_{p}\left(f_{i},t\right)\;\cdot \;e^{-j2\pi f_{i}\tau_{p}\left(t\right)},\;i\in \left[1,N_{\mathrm{sub}}\right].$$

**Figure 1:**
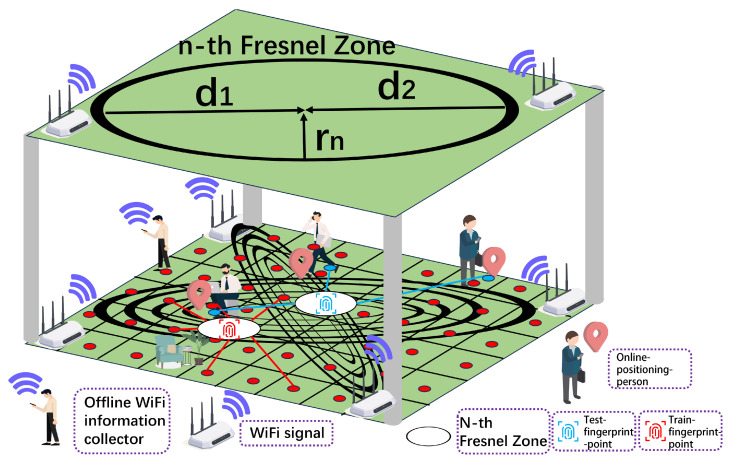
The detailed model diagram.

In the frequency domain, the above H(f,t) turns into $H_{i,j,N_{sub},t}$. It represents each CSI data packet, where *i* and *j* denote the transmitting and receiving antennas. In this experiment, there is only one transmitting and one receiving antenna to simplify $H_{i,j,N_{sub},t}$ into a two-dimensional matrix $H_{N_{sub},t}$. The theoretical scene diagram and the wave transmission are shown in Figure 1. The $N_{sub}$ represents the sub-carrier number, and the CSI matrix is shown as follows

(2) $$\;\mathrm{CSI}\;\mathrm{matrix}\;=\left[\begin{array}{cccc} H_{1,1} & H_{1,2} & \cdots & H_{1,t}\\ H_{2,1} & H_{2,2} & \cdots & H_{2,t}\\ \vdots & \vdots & \ddots & \vdots \\ H_{N_{\mathrm{sub}},1} & H_{N_{\mathrm{sub}},2} & \ldots & H_{N_{\mathrm{sub}},t} \end{array}\right].$$

The Fresnel Zone is described as the shape of an ellipse of wireless communications. According to the distance between the transmitter and receiver points, the different shapes of the Fresnel Zone can be used as the indicator of the fingerprint points. For the Nth Fresnel Zone and electromagnetic waves with wavelength $\frac{\lambda }{2}$, the distance of the ellipse can be described as follows.

(3) $$N\frac{\lambda }{2}=\sqrt{d_{1}^{2}+r_{n}^{2}}+\sqrt{d_{2}^{2}+r_{n}^{2}}-\left(d_{1}+d_{2}\right).$$

Due to the ellipsoidal shape of the Fresnel Zone, any arbitrary point is located at a distance $d_{1}$ from the transmitter (TX) and $d_{2}$ from the receiver (RX). Each Fresnel Zone has a unique radius, denoted as $r_{n}$. The detailed model diagram is shown in Figure 1.

## 4. Proposed Data Processing and NKCK Method

This section outlines the indoor positioning sensing strategy, which consists of three components: multiple independent transmission and collection links, optimized preprocessing, and the modeling process. The approach employs a fingerprint method, divided into offline and online phases, during which the data are processed as training and testing sets.

### 4.1. Multiple Transmission and CSI Collection Links

The system consists of multiple independent transmission and collection links designed for experimental device layout and data collection processes. The devices are made up of both software and hardware modules. The software modules include nexus monitor (Nexmon), which patches firmware, and a Python program that establishes a transmission control protocol/internet protocol (TCP/IP) socket connection. On the hardware side, the system utilizes a Broadcom 2711B0 processor found in multiple Raspberry Pi 4B devices, along with a single AP for connection. The collection hardware features a Broadcom 2837B0 processor with a BCM43455C0 WiFi module, based on multiple Raspberry Pi 3B+ units. The AP operates in the 5GHZ frequency band, with a bandwidth of 40 MHZ, and 157 channels, and supports the 802.11a/c protocol. During the connection process, the AP assigns an IP address to each Raspberry Pi 4B.

The Raspberry Pi 4B establishes a socket connection with the AP, sending data every 400 milliseconds via the Python program. Meanwhile, the collection module activates monitoring mode to gather CSI data. The AP’s channel and bandwidth settings, as well as the MAC address of the Raspberry Pi 4B, are configured by the Raspberry Pi 3B+, which receives a Base64 encoded parameter string. This parameter string is then used to configure the CSI extractor.

The Raspberry Pi 3B+ also enables monitoring mode and collects CSI by listening to the user datagram protocol (UDP) socket on port 5500, using tcpdump—a tool that captures network traffic. In this study, the sampling rate is set to 50 packets per second, with a total collection time of 360 s, resulting in 18,000 sampling packets. Every 50 packets are stored in a packet capture (Pcap) file, which includes a Pcap Header, Packet Header, and Packet Data. The Packet Data contain the original CSI data.

### 4.2. Preprocessing of CSI Data

According to the Pcap files from the collection links, each Pcap file is parsed to extract sampling packets of the original complex data using specific firmware. Based on the 802.11a/c protocol [30], 128 subcarriers are used to transmit the original complex data. Out of these, only 108 subcarriers contain the actual data information, while certain edge and middle subcarriers do not provide effective CSI. Additionally, some subcarriers are designated as pilot subcarriers, which aid in channel tracking. Their indexes are ±11, ±25, and ±53. The null subcarriers at direct current (DC) are included to ensure the spectrum decays gracefully and meets spectral mask specifications. Their indexes are −64, ±63, ±62, ±61, ±60, ±59, ±1, and 0. As a result, the pilot and DC subcarriers are removed, leaving the remaining subcarriers to carry the original CSI data.

After completing the previous steps, the original CSI data are extracted from each Pcap file. The preprocessing process is divided into two key aspects, as outlined below.

Firstly, from the perspective of the preprocessing procedure, we focus on selecting the principal waveform and extracting ideal amplitude data. In the principal waveform selection step, PCA is used in conjunction with DBSCAN clustering to process the extracted amplitude data. Since disruption at different positions can lead to a specified fingerprint point containing the waveforms of other unknown positions, PCA is chosen for its capability as an unsupervised dimensionality reduction method. Once the Pcap files are reduced to lower dimensions, DBSCAN helps distinguish between different categories. It is important to note that the remaining data consist of Pcap files corresponding to the principal waveform. The PCA and DBSCAN methods aim to filter out Pcap files with abnormal waveforms. After selecting the principal waveform, a mean filter based on the sliding window algorithm is applied to obtain ideal CSI amplitude data, which include 108 data subcarriers in the CSI data preprocessing system.

**Figure 2:**
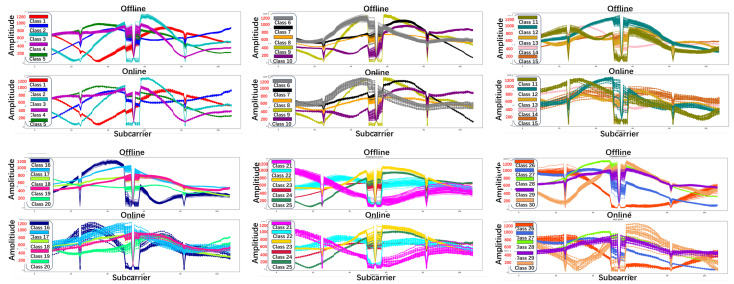
The offline and online CSI amplitude waveform graph of 30 fingerprint points after preprocessing.

Secondly, from the perspective of the fingerprint process, fingerprint preprocessing is divided into two stages: offline and online. In the offline stage, all collected Pcap files are processed using PCA for dimensionality reduction and DBSCAN clustering with an optimized sliding window algorithm. The results serve as a training set for the novel method developed in this paper. A total of 80 Pcap files, each representing a specific fingerprint point, are selected and organized into a two-dimensional array with 108 rows and 80 columns. In the online stage, we analyze the variance between the online Pcap files and the preprocessed offline training set. From this analysis, we select 20 suitable Pcap files corresponding to each fingerprint point to create a testing set. The training and testing sets for the 30 fingerprint points are illustrated in Figure 2.

### 4.3. NKCK Method Based on Machine Learning

**Figure 3:**
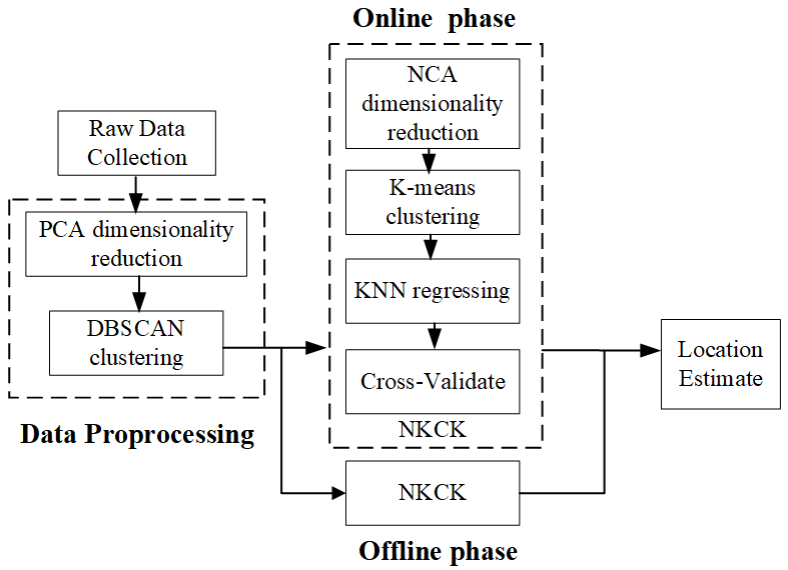
Offline and online collection flowchart.

After preprocessing, the CSI data are divided into testing and training sets. To summarize various learning methods, an approach called NKCK is proposed here, which incorporates NCA for dimensionality reduction, K-means clustering, and KNN classification. Additionally, we introduce the CV method to evaluate the KNN classification model. This method includes the NCA dimensionality reduction, K-means clustering, and KNN classification processes. NCA [31] enhances feature selection by calculating the Mahalanobis distance [32] across different feature sets, focusing on interclass correlations and reducing sample sets. The offline and online flowchart is illustrated in Figure 3.

#### 4.3.1. NCA Dimensionality Reduction

The CSI amplitude data *s* belong to the CSI amplitude dataset $S^{p\times h}$, where h=n×m, *n* represents the number of fingerprint points, and *m* denotes the number of CSI data packets in each fingerprint point. Since the CSI amplitude is on a 5GHz frequency, a 40 MHZ bandwidth channel is obtained at the receiving end, and hence p=128.

The mean of CSI amplitude data on the *i*-th fingerprint point is denoted by $\bar{s_{i}}$, and the mean of CSI amplitude data on *j*-th all fingerprint points is denoted by ${\bar{s}}_{j}\in S^{p\times h}$. *M* is symmetric positive semidefinite, and *T* represents the transpose of the matrix. There is a supposed mapping matrix $A\in R_{k\times d}$,k<d,k≥rank(M). The Mahalanobis distance $d_{M}\left(\bar{s_{i}},{\bar{s}}_{j}\right)$ will be defined as follows

(4) $$d_{M}\left({\bar{s}}_{i},{\bar{s}}_{j}\right)=\sqrt{{\left({\bar{s}}_{i}-{\bar{s}}_{j}\right)}^{T}M\left({\bar{s}}_{i}-{\bar{s}}_{j}\right)},$$

(5) $$d_{M}\left({\bar{s}}_{i},{\bar{s}}_{j}\right)=\sqrt{{\left({\bar{s}}_{i}-{\bar{s}}_{j}\right)}^{T}A^{T}A\left({\bar{s}}_{i}-{\bar{s}}_{j}\right)}={∥A{\bar{s}}_{i}-A{\bar{s}}_{j}∥}_{2}.$$

Let $p_{ij}$ be defined as the neighbor’s probability distribution of i-th sample

(6) $$p_{ij}=\frac{\exp(-∥A{\bar{s}}_{i}-A{\bar{s}}_{j}{∥}^{2})}{\sum_{k\neq i}\exp(-∥A{\bar{s}}_{i}-A{\bar{s}}_{k}{∥}^{2})},\;p_{ii}=0.$$

The optimization goal is constructed by f(A), which corresponds to the expected number of points correctly classified under the probability distribution

(7) $$f\left(A\right)=\sum_{i=1}^{n}p_{i}=\sum_{i=1}^{n}\sum_{j\in S^{p\times h}}p_{ij}.$$

To obtain the maximum value of the objective, the process calculates the gradient of the optimization objective. The mapping matrix *A* is obtained through the gradient descent method. The $S^{p\times h}$ can reduce the dimensionality of $S^{l\times h}$ by reflecting into the *A* matrix, and *p* is deduced into low dimensions *l*

(8) $$\frac{\partial f}{\partial A}=-2A\sum_{i}\sum_{j\in S^{p\times h}}p_{ij}\left(s_{ij}s_{ij}^{⊤}-\sum_{k}p_{ik}s_{ik}s_{ik}^{⊤}\right),$$

(9) $$\begin{array}{cc} \frac{\partial f}{\partial A} & =2A\sum_{i}\left(p_{i}\sum_{k}p_{ik}s_{ik}s_{ik}^{⊤}-\sum_{j\in S^{p\times h}}p_{ij}s_{ij}s_{ij}^{⊤}\right) \end{array}.$$

The dimensionality reduction method is applied to extract the principal components. Compared to unsupervised methods like PCA, supervised methods are more suitable for this system. Among the supervised dimensionality reduction techniques, NCA, which utilizes the Mahalanobis distance, proves to be more effective at identifying similarities among fingerprint points than alternatives such as LDA.

The specific procedure for dimensionality reduction is as follows: In the Python environment, a two-dimensional array is used to store the training and testing sets. After applying the NCA dimensionality reduction process, the training sets, which contain 80 samples of 30 fingerprint points across 108 dimensions, are reduced to a new set with 80 rows and 3 dimensions. The testing sets are similarly reduced to three dimensions. Furthermore, this three-dimensional representation, based on spatial vectors, retains more information than other dimensional formats and is more effective for processing the 30 fingerprint points.

#### 4.3.2. K-Means Clustering with Elbow Method

When performing dimensionality reduction, the data should be labeled according to their corresponding category once the dimensions are reduced. Points that are far from the category center are identified as anomalous data in DBSCAN. K-medoids [33] is not effective at addressing these distant points. In contrast, K-means clustering is more effective at identifying outliers in three-dimensional space related to CSI data compared to other clustering methods such as DBSCAN and K-medoids. Prior to clustering, the optimal K value for K-means is determined using the Elbow Method.

In the K-means process, the CSI amplitude dataset after NCA dimensionality reduction is expressed as $S^{l\times h}$. The *K* cluster center corresponds to the fingerprint point number, and *l* denotes the dimensions of each data sample. $d(s_{i},s_{j})$ represents the distance between the center and other sample data. The K-means method calculates Euclidean distance as follows

(10) $$d\left(s_{i},s_{j}\right)=\sqrt{\left(s_{i}-s_{j\in S^{l\times h}}\right){\left(s_{i}-s_{j}\right)}^{T}}.$$

The distance value update follows the center in an iterative process. $C_{k}$ and $|C_{k}|$ represent the *i*-th cluster center and data object number, respectively. The following algorithm calculates this center value

(11) $$Center_{k}=\frac{1}{|C_{k}|}\sum_{s_{i}\in C_{k}}s_{i}.$$

The process is circled until the total distance error is less than the present threshold δ

(12) $$\Delta J=\sum_{k=1}^{K}\sum_{s_{i}\in C_{k}}d\left(s_{i},Center_{k}\right).$$

#### 4.3.3. KNN Classification with CV

When the cluster finishes, KNN classification builds the model by calculating the distance between samples. The distance algorithm is calculated as follows

(13) $$r=d\left(s_{i},s_{j}\right)=\sqrt{\sum_{i=1}^{n}{\left[a_{r}\left(s_{i}\right)-a_{r}\left(s_{j}\right)\right]}^{2}}.$$

where $a_{r}\left(s\right)$ represents the *r*-th attribute value of the $s_{i}$ and $s_{j}$ samples.

The KNN algorithm is used to model the training sets and classify the testing sets. Before modeling, CV is employed to determine the optimal K value for KNN classification. This study utilizes Shuffle Split, K-fold, Stratified K-fold, and Leave-p-out methods to evaluate the K value. The experimental results indicate that Shuffle Split was chosen as the basis for performance evaluation. For the Shuffle Split method, the parameters are configured for 2 splits, with 2400 training samples and 600 testing samples, utilizing 79 random states.

### 4.4. Comparison Performance

Here, we consider to use three positioning evaluation functions: root mean square error (RMSE), mean square error (MSE), and mean absolute error (MAE). The variable N denotes the number of fingerprint points. The mathematical expressions for these functions are presented below.

(14) $$\mathrm{RMSE}=\sqrt{\left(\frac{1}{N}\sum_{i=1}^{N}\left[{\left(x_{i}-x_{i}^{\prime }\right)}^{2}+{\left(y_{i}-y_{i}^{\prime }\right)}^{2}\right]\right)},$$

(15) $$\mathrm{MSE}=\left(\frac{1}{N}\sum_{i=1}^{N}\left[{\left(x_{i}-x_{i}^{\prime }\right)}^{2}+{\left(y_{i}-y_{i}^{\prime }\right)}^{2}\right]\right),$$

(16) $$\mathrm{MAE}=\left(\frac{1}{N}\sum_{i=1}^{N}\left(\left|x_{i}-x_{i}^{\prime }\right|+\left(∥y_{i}-y_{i}^{\prime }\right)|\right)\right).$$

## 5. Performance Evaluation

A uniform distribution fingerprint point was applied to the multi-user position in the paper. The fingerprint points were arranged in triangular, random, and radial distributions. In the triangular distribution, points form a triangular pattern, while positioning accuracy decreases near the boundaries due to channel fading. Random distribution cannot guarantee consistent positioning accuracy. The radial distribution mimics the channel fading pattern, while points closer to the signal source tend to interfere with each other.

**Figure 4:**
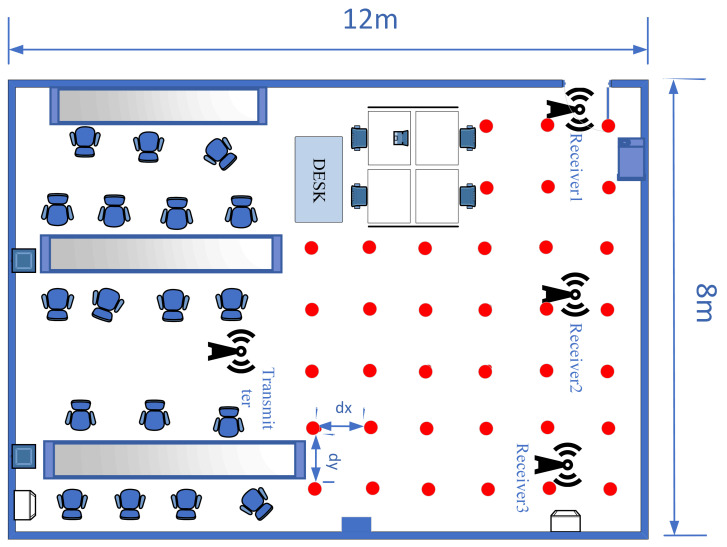
The laboratory layout.

**Figure 5:**
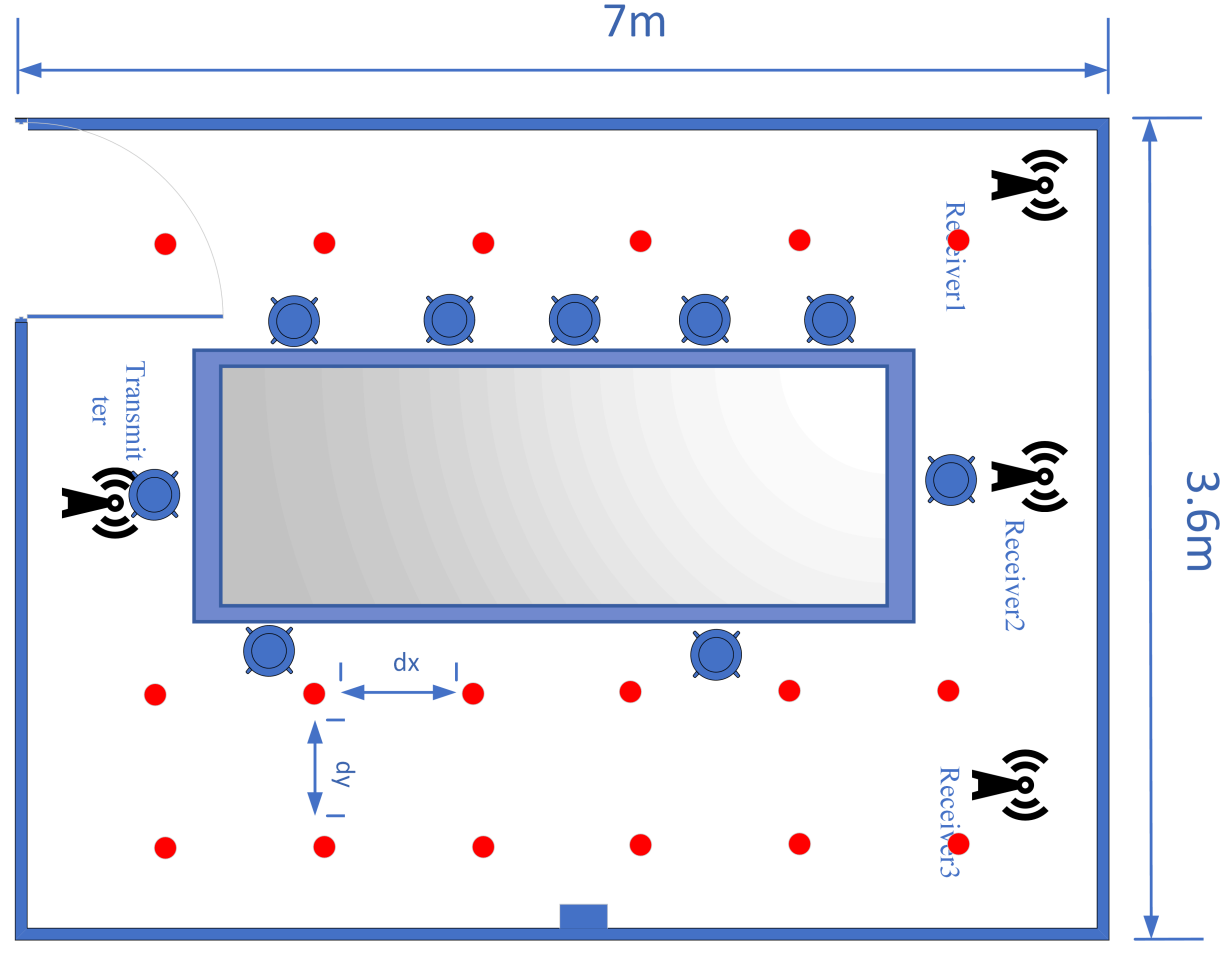
The meeting room layout.

**Figure 6:**
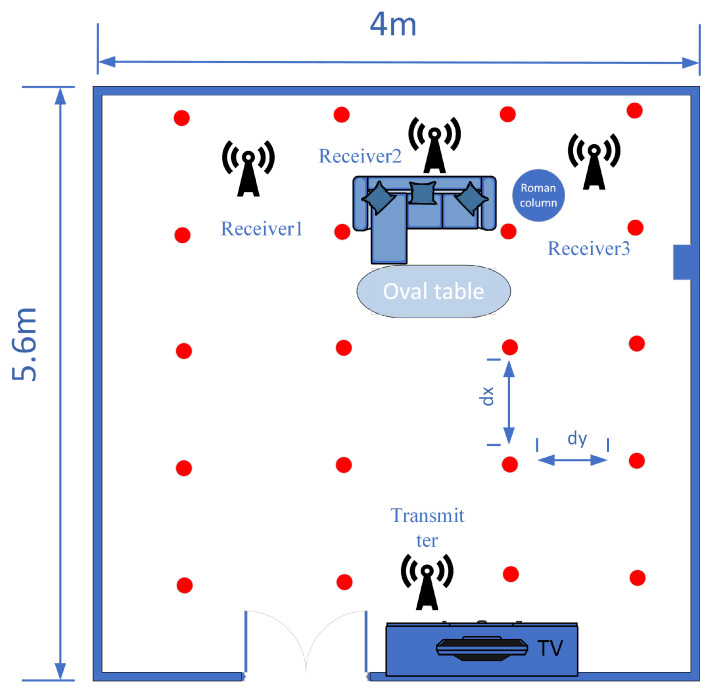
The living room layout.

The study found that a uniform distribution of fingerprint points yielded the best localization results; therefore, it was used to arrange the fingerprint points. Reference points and test points are grouped based on their distances. Fingerprint points that are too close together can interfere with each other’s signal transmission and reception, leading to decreased localization accuracy. Conversely, fingerprint points that are too far apart may also reduce accuracy due to lower fingerprint density. The experimental results helped determine the optimal distance between fingerprint points, as indicated in Figure 4, Figure 5 and Figure 6.

In the next phase, we will discuss the influence of three different scenarios on this system. The performance of the NKCK method will be analyzed in the contexts of a laboratory, meeting room, and house. Following this, the performance of the NKCK method will be compared to other fingerprint positioning methods. Lastly, we will assess the impact of varying numbers of online users in the laboratory setup, which features three APs and a Raspberry Pi that are precisely positioned. During the offline phase, a person will stand at each fingerprint point sequentially to collect the training set. In the online phase, test points will be chosen randomly to gather the testing set. Finally, the data will be verified for positioning accuracy according to the fingerprint training model.

### 5.1. Experimental Scenarios

Here, we examine the impact of different experimental environments on our system, specifically focusing on horizontal and longitudinal distances (dx and dy). We particularly analyze how the robustness of our multi-user localization system compares to the public accuracy in the conference room. For example, we observe that varying degrees of signal fading can affect performance in each scenario. In the meeting room, a rectangular table alters reflection paths, which subsequently reduces positioning accuracy.

These observations highlight how environmental factors, such as obstacles, can influence the performance of our method in different scenarios. This paper will further elaborate on this, clarifying how the method adapts to various environments and how these factors contribute to the results we observe.

#### 5.1.1. Laboratory

In this subsection, the area of a specific scientific research laboratory is 12 m × 8 m, and dx and dy are equal to 2 m, respectively. In the offline stage, the Raspberry Pi 4B is set in sequence at the specified position, and the router with Raspberry Pi 3B+ is placed far away from the fingerprint point. Meanwhile, several positions are randomly regarded as the testing point in the online stage. The detail is shown in Figure 4.

#### 5.1.2. Meeting Room

Compared to the laboratory environment, a meeting room condition is more complex. A typical meeting room is the second experimental scene. Its covered area is 7 m × 3.6 m, and dx and dy are both 1.2 m, approximately. The 18 fingerprint points are shown in Figure 5.

The room’s geometry, including the large rectangular meeting table and the surrounding desks (as shown in Figure 5), influences signal propagation by introducing multiple reflective surfaces and obstructions. The table, for example, reflects the transmitted signal, which can affect the propagation paths and cause variations in localization accuracy. Additionally, the room’s relatively controlled space size and fewer reflective surfaces compared to other environments, such as a house, result in different signal fading and reflection patterns. These factors contribute to the differences observed in performance compared to the laboratory environment.

#### 5.1.3. House

The final scenario takes place in a living room, which presents more complexity than the previous two settings. Various fixed furniture items, such as tables, air conditioners, and marble pillars, are positioned in every corner of the room. The covered area measures approximately 5.6 m × 4 m, with dx and dy set to 0.8 m. Additionally, 12 fingerprint points are illustrated in Figure 6.

The irregular layout and the presence of large objects—like the sofa and TV unit—result in signal variations due to interference and reflections from these obstacles. The differing reflection and absorption of WiFi signals, influenced by the materials and arrangement of the space, lead to decreased positioning accuracy. This is because the collection of CSI data is affected by these factors.

**Figure 7:**
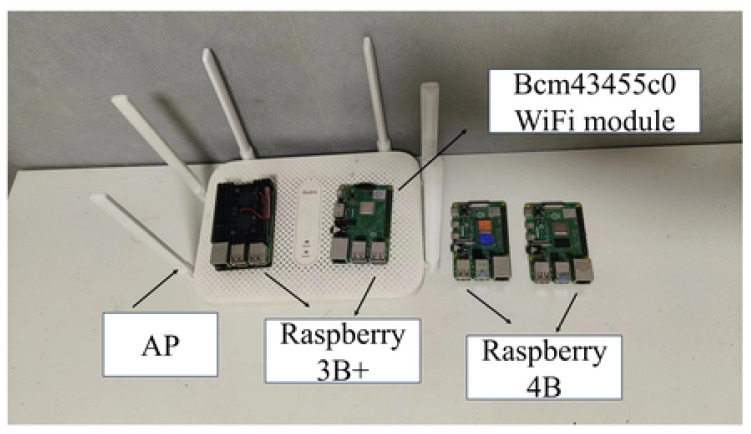
The system of our experiment.

Analyzing the CSI data in such environments reveals the challenges posed by signal disruption from both the irregular layout and larger obstacles. The experimental system is depicted in Figure 7, which includes three Raspberry Pi 3B+ devices equipped with bcm43455c0 WiFi cards as CSI receivers, along with a Redmi commercial router. This setup works with the three Raspberry Pi 3B+ devices, utilizing a bandwidth of 40 MHZ and operating on channel 157 as the signal transmitter. In the experiments, the three Raspberry Pi 3B+ used three receiving antennas, while three Raspberry Pi 4B utilized three transmitting antennas.

### 5.2. Performance Evaluation

RMSE was utilized to assess the performance of the method. Initially, the RMSE for NKCK was evaluated within a specific environment, incorporating PCA for preprocessing the CSI data and SVM for position prediction [19]. This evaluation included various approaches such as indoor LDA-based CSI amplitude fingerprinting (LCAF) [22], CSI indoor localization using DBSCAN clustering [26], random forest (RF) [34], multilayer perceptron (MLP) [35], naive Bayes (NB) [36], and SVM [37].

**Figure 8:**
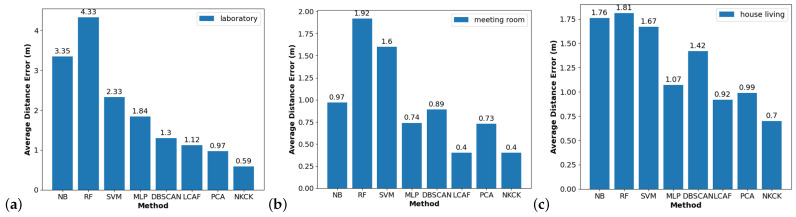
RMSE of different methods.

The laboratory results are presented in Figure 8a. The NKCK method achieved the lowest RMSE of 0.59 m. In comparison, the RMSEs for the other methods—NB, RF, SVM, MLP, DBSCAN-based, LCAF, and PCA-based—were 3.35 m, 4.33 m, 2.33 m, 1.84 m, 1.3 m, 1.12 m, and 0.97 m, respectively. This means that NKCK outperformed these methods by gains of 82.4%, 85.0%, 72.1%, 64.7%, 50.0%, 42.0%, and 33.0%.

Figure 8b,c display the results from other scenarios. Overall, these findings demonstrate the robustness of the NKCK method across the three scenarios. In the meeting room, the NKCK method’s gains compared to NB, RF, SVM, MLP, and DBSCAN-based methods were 58.8%, 79.1%, 75.0%, 45.9%, and 55.1%, while the gains for LCAF and PCA-based methods were 0% and 45.2%, respectively. In the house, the performance gains compared to NB, RF, SVM, MLP, and DBSCAN-based methods were 60.2%, 61.3%, 58.1%, 50.7%, and 34.6%, respectively, alongside gains of 23.9% and 29.3% for LCAF and PCA-based methods.

**Figure 9:**
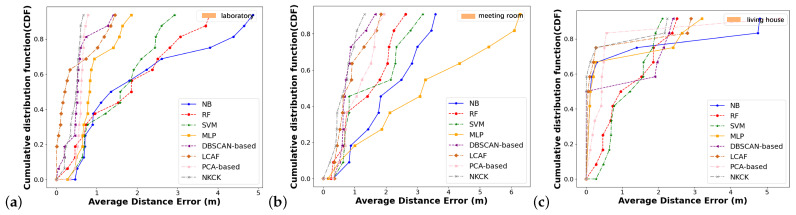
CDFs of localization error of different scenarios in an AP.

To assess data characteristics in our scenarios, we utilized cumulative distribution functions (CDF) and box plots. Figure 9a illustrates the CDFs of distance errors in the laboratory. The CDF for the NKCK method reaches 80% at 1.12 m, whereas the CDFs for the other methods—DBSCAN-based, LCAF, PCA-based, RF, SVM, NB, and MLP—are 1.45 m, 1.82 m,1.82 m, 2.52 m, 3.01 m, 3.64 m, and 6.5 m, respectively. Figure 9b describes the CDFs in the meeting room. The CDF for the NKCK method reaches 80% at 1.34 m, whereas the CDFs for the other methods—NB, RF, SVM, MLP, LCAF, and PCA-based—are 1.82 m, 1.94 m, 1.96 m, 2.76 m, 3.96 m, and 6.52 m, respectively. Figure 9c shows the case of the CDFs of distance errors in the house. The CDF for the NKCK method reaches 75% at 0.47 m, whereas the CDFs for the other methods—NB, RF, SVM, MLP, LCAF, and PCA-based—are 0.49 m, 0.72 m, 1.56 m, 1.96 m, 1.99 m, and 2.23 m, respectively.

**Figure 10:**
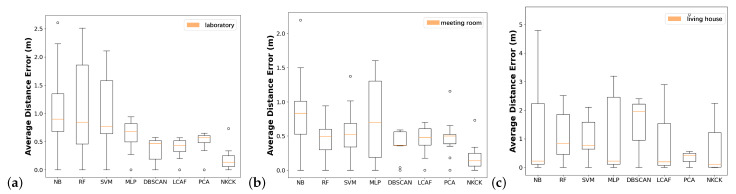
The box plot of localization error of different scenarios in an AP.

To analyze the accuracy of different methods, we used quartile charts. Figure 10a presents the box plot for the laboratory results. We conclude that the upper quartile, median, and lower quartile of our proposed method were the smallest. Figure 10b illustrates the findings from the meeting room. It can be found that our proposed method has the smallest upper, median, and lower quartiles. Figure 10c shows the box plot in the house. We can find that the median and lower quartiles are the smallest in the NKCK method. In conclusion, the NKCK method proves to be a suitable addition to this indoor positioning system.

**Figure 11:**
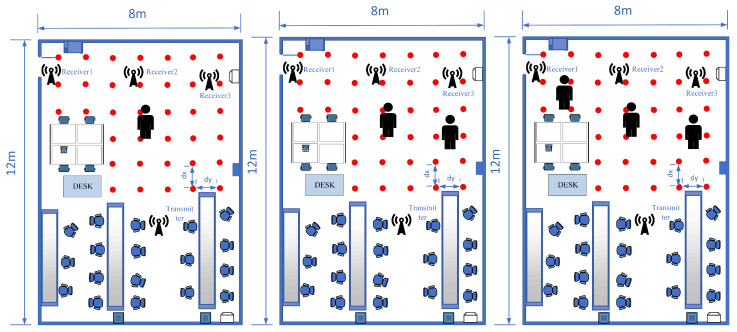
Positioning error of different numbers of users in an AP.

**Figure 12:**
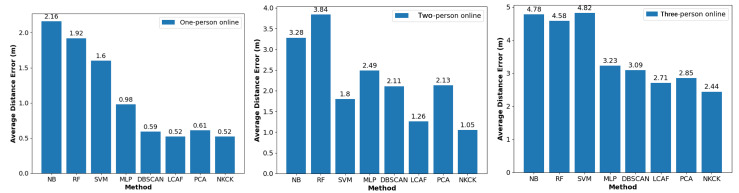
Localization error of different methods in an AP.

In addition, the impact of the number of online users on positioning performance was explored in the laboratory, with results shown in Figure 11 and Figure 12. For the case of one user online, the proposed NKCK method achieved error reductions of 76.6% compared to NB, 72.9% compared to RF, 67.5% compared to SVM, 46.9% compared to MLP, 11.9% compared to DBSCAN-based methods, and 14.8% compared to PCA-based approaches. In the case of two users online, the NKCK method reduced errors by 68%, 72.7%, 41.7%, 57.8%, 50.2%, and 50.7%, and in the case of three users online, by 49.0%, 46.7%, 49.4%, 24.5%, 21.0%, and 14.4%, respectively, when compared to the same methods. In the CDF chart, for an RMSE of 2m, our proposed method achieved 89.3% accuracy, compared to LCAF’s 57.7%. The RMSE of the NKCK method increased from 0.52 m to 1.05 m and then to 2.44 m as the number of users grew.

**Figure 13:**
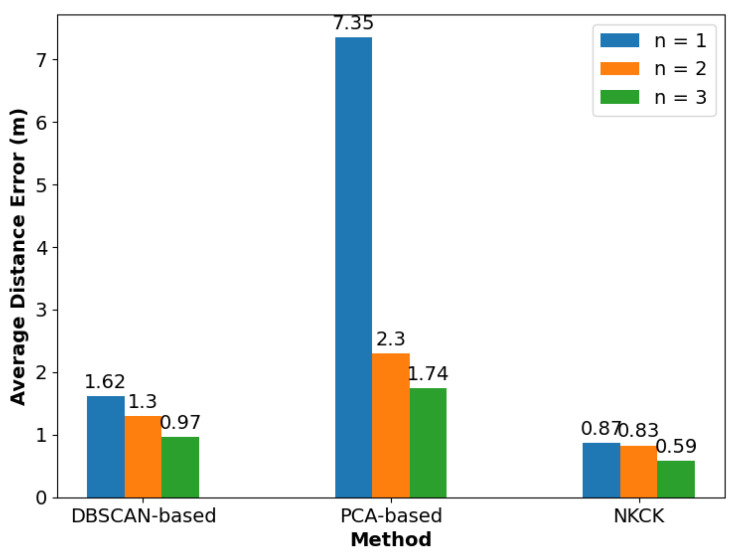
RSME for different methods with different numbers of reference points.

In this experiment conducted in the laboratory, various numbers of APs were utilized. Notably, the experiment excluded the algorithms NB, MLP, SVM, RF, and LCAF, as their strategies do not support the use of multiple APs. Figure 13 illustrates the different RMSE values for three localization methods based on the number of APs used. In Figure 13, the DBSCAN-based method shows an increase in RMSE: 0.65 m with one AP and 0.33 m with two APs, when compared to the use of three APs. Similarly, the PCA-based method exhibits a more substantial increase of 5.05 m with one AP and 0.56 m with two APs under the same conditions. In contrast, our proposed NKCK method demonstrates only a slight increase in RMSE of 0.28 m with one AP and 0.24 m with two APs compared to three APs. These results indicate that the number of APs does not significantly impact the robustness of the NKCK strategy.

## 6. Conclusions

This paper presented a novel strategy for processing CSI data, aimed at improving the accuracy of distinguishing neighbors’ positions and predicting their online locations. A primary challenge is the computational complexity associated with processing large volumes of CSI data, especially when scaling the system to environments with higher user density. As the number of users and APs increases, the demand for computational resources may rise significantly, which can impact real-time processing speeds and overall system efficiency. Additionally, in high-density settings, overlapping CSI signals can complicate the accurate identification of closely positioned users, indicating another area that requires future optimization. Our results show that the system can effectively localize at least three users with an average accuracy of 1.26 m.

## Figures and Tables

**Table 1:** Comparison of different models.

Author	Method	Features	Scenario	Results	Cons	Multi-User (Yes/No)
Ours	NKCK	CSI	Lab. 12 × 8 m^2^ Meeting room 7 × 3.6 m^2^ House 5.6 × 4 m^2^	1.26 m	**three** **links** **(Pros)**	**Yes**
[19]	PCA SVM	CSI	Lab. (3.6 × 3.6 m^2^)	0.9 m	single link	No
[20]	PCA DWT	CSI	Classroom	1.48 m	multi- link	No
[21]	MDWT	CSI	Room (140 m^2^)	1.5 m	single link	No
[22]	LDA	CSI	Room	human activity	single link	No
[23]	KNN	CSI	Room (12 × 15 m^2^)	1.7 m	single link	No
[24]	DWT PCA SVM	CSI	Room	activity (96.6%)	single link	No
[25]	broad learning	CSI	Lab. (13.5 × 11 m^2^)	1.73 m	single link	No
[26]	PCA DBSCAN	CSI	Lab. 7 × 6 m^2^ Meeting room 6 × 6 m^2^	>1.22 m <1.39 m	single link	No
[27]	ALS algorithm	CSI	Lab. (42.08 × 3.12 m^2^) Lab. (22.72 × 8.04 m^2^)	3 m	single link	No
[28]	AdaBoost	CSI	Room (6 × 7 m^2^)	<2 m	single link	No
[29]	DFPhaseFL	CSI	Room (12 × 10 m^2^)	1.0 m	single link	No

## Data Availability

Data are contained within the article.
